# Effect of video angle on detection of induced front limb lameness in horses

**DOI:** 10.1186/s12917-024-04032-9

**Published:** 2024-05-03

**Authors:** Alessandro P. Valle, Kara A. Brown, Patrick Reilly, Sarah A. Ciamillo, Elizabeth J. Davidson, Darko Stefanovski, Holly L. Stewart, Kyla F. Ortved

**Affiliations:** Department of Clinical Studies, New Bolton Center, University of Pennsylvania, 382 West Street Road, Kennett Square, Philadelphia, PA 19348 United States of America

**Keywords:** Lameness, Video, Telemedicine, Lameness locator

## Abstract

**Background:**

Lameness examinations are commonly performed in equine medicine. Advancements in digital technology have increased the use of video recordings for lameness assessment, however, standardization of ideal video angle is not available yielding videos of poor diagnostic quality. The objective of this study was to evaluate the effect of video angle on the subjective assessment of front limb lameness. A randomized, blinded, crossover study was performed. Six horses with and without mechanically induced forelimb solar pain were recorded using 9 video angles including horses trotting directly away and towards the video camera, horses trotting away and towards a video camera placed to the left and right side of midline, and horses trotting in a circle with the video camera placed on the inside and outside of the circle. Videos were randomized and assessed by three expert equine veterinarians using a 0–5 point scoring system. Objective lameness parameters were collected using a body-mounted inertial sensor system (Lameness Locator®, Equinosis LLC). Interobserver agreement for subjective lameness scores and ease of grading scores were determined.

**Results:**

Induction of lameness was successful in all horses. There was excellent agreement between objective lameness parameters and subjective lameness scores (AUC of the ROC = 0.87). For horses in the “lame” trials, interobserver agreement was moderate for video angle 2 when degree of lameness was considered and perfect for video angle 2 and 9 when lameness was considered as a binary outcome. All other angles had no to fair agreement. For horses in the “sound” trials, interobserver agreement was perfect for video angle 5. All other video angles had slight to moderate agreement.

**Conclusions:**

When video assessment of forelimb lameness is required, a video of the horse trotting directly towards the video camera at a minimum is recommended. Other video angles may provide supportive information regarding lameness characteristics.

**Supplementary Information:**

The online version contains supplementary material available at 10.1186/s12917-024-04032-9.

## Background

Lameness examinations are the primary method used by equine veterinarians to localize sources of pain and decreased performance in horses. During lameness examinations, horses are often evaluated trotting in a straight line and while circling, as circling can exacerbate lameness [1]. Traditionally, lameness examinations are performed by the attending veterinarian in person, either at the farm or at an equine clinic. With advancements in digital technology and telemedicine, video recordings of horses moving at different gaits in hand and under saddle are increasingly provided to equine veterinarians for assessment. Horse owners and trainers often send video recordings to veterinarians for triage purposes, as part of prepurchase examinations (PPE), and to keep veterinarians appraised of response to therapy and rehabilitation progress. Veterinarians also commonly video record lameness examinations for later review, for consultation with associates, and for educational purposes.

Previous studies have provided conflicting results on the accuracy of lameness assessment using video recordings [2–5]. Hardeman et al. (2022) demonstrated small differences in the accuracy of lameness evaluation of horses trotting in a straight line performed in person or via video recording [2], while Rungsri et al. (2014) found significantly better agreement in detection of forelimb lameness when horses were examined in person (high agreement) versus by video recording (fair to moderate agreement) [5]. Video assessment of hind limb lameness has also yielded low interobserver agreement [6], however, Fuller et al. (2006) found acceptable reliability of interobserver lameness assessment using video recordings of horses with fore and hind limb lameness with the sound of the horse trotting included on the video [3].

Despite the conflicting results regarding video assessment of lameness, refinement of video recording of horses at the trot for the purpose of lameness evaluation may be possible and could enhance the utility of telemedicine in equine practice and benefit future research studies utilizing video recordings. Currently, equine veterinarians are asked to review video recordings of horses that are acquired from a variety of angles and positions, many of which are not of diagnostic quality. High quality, diagnostic videos could allow veterinarians to assess lameness when live examinations are not possible due to scheduling or transportation limitations, or to assess response to treatment and rehabilitation. In addition, video recordings would negate the need for repeated stresses associated with transportation, decrease potential pathogen exposure, and limit introduction to unfamiliar people and animals [7]. Thus, an ideal video protocol is necessary to provide to owners, trainers, and veterinarians that are video recording horses for evaluation purposes.

Although previous studies have compared live lameness assessment to video assessment, no studies have examined the effect of video angle on lameness assessment. As there are many factors affecting subjective visual examinations, the objective of this study was to examine the effect of video angle on detection of forelimb lameness. We hypothesized that video recordings of horses trotting in a straight line away and towards the observer would be the most accurate video angle for detection of forelimb lameness.

## Results

### Video trials

A total of 108 video trials were available for review by the 3 equine veterinarians board-certified in equine sports medicine and rehabilitation (KFO, KAB, EJD). Each horse had a video recording of a “sound trial” i.e. no lameness induced, and “lame trial” i.e., lameness induced, using each of the 9 video angles. The video angles included: (1) straight away, (2) straight towards, (3) straight away and towards, (4) left away and towards, (5) right away and towards, (6) inside left circle, (7) inside right circle, (8) outside left circle, (9) outside right circle. No trials were excluded from the analysis.

### Induction of lameness

**Fig. 1:**
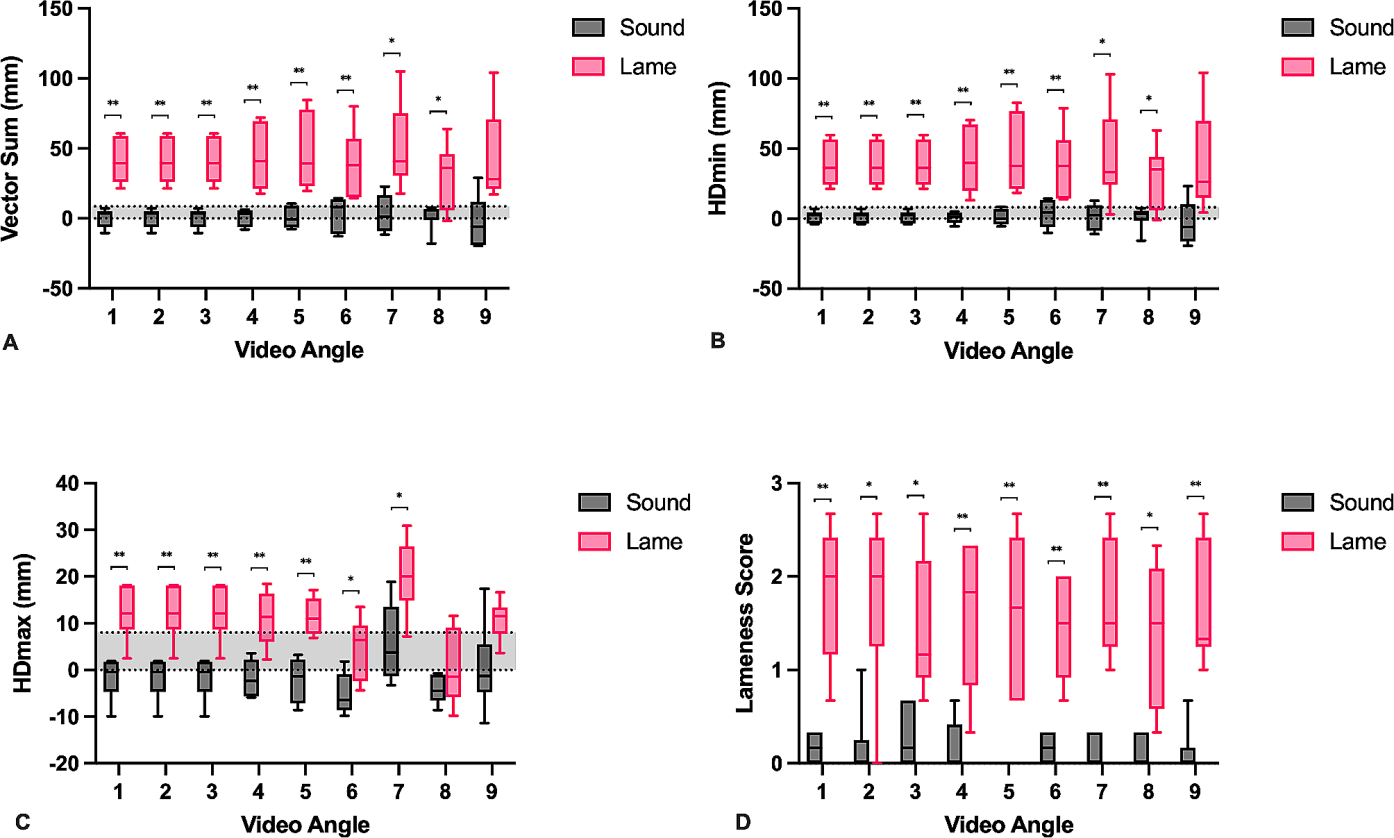
Box plots of the **(A)** vector sum, **(B)** minimum head displacement (HDmin), **(C)** maximum head displacement (HDmax), and **(D)** subjective lameness scores in sound horses and horses with induced lameness being assessed at 9 video angles. Box and whisker plots represent median (center line), 25% and 75% percentiles (box end lines), and minimum and maximum (whiskers). Shaded areas in figure **A**), **B**), and **C**) represent vector sum, HDmin and HDmax in which horses are considered sound. * *p* < 0.05, ** *p* < 0.01

Induction of lameness was successful as determined by subjective lameness scores and objective lameness parameters (Fig. 1). The median (range) of strides included for analysis was 22 (15–29). Vector sum (VS; mm), difference in head minimum heights (HD_min_; mm), and difference in head maximum heights (HD_max_; mm), and subjective lameness scores were all significantly increased in horses with induced lameness. The only exception to this were horses in video angle 8 (outside left circle) and 9 (outside right circle) which had an increased HD_max_, however, the difference was not statistically significant compared to the sound trials. Overall, mean VS(mm) was > 8.5 mm in all horses with induced lameness in all video angles which is considered to be above the threshold for forelimb lameness [8, 9]. When all angles were considered together, horses that were recorded as having a 1/5 lameness were 66.6 times more likely to have had lameness induced in that limb than not (95% CI 20.0-221.4; *p* < 0.0001), while horses that were recorded as having a 2/5 lameness were 3547.7 times more likely to have had lameness induced in that limb than not (95% CI 287.6-43756.9; *p* < 0.0001). Overall, there was excellent agreement between objective lameness parameters and subjective lameness scores (AUC of the ROC = 0.87, 95% CI: 0.83–0.90). The effect of limb on detection of lameness during video angles of horses trotting in a circle was also examined as lameness can often be exacerbated with the lame limb on the inside or outside of the circle. No significant effect of limb was found on lameness detection when video recordings were obtained from the inside or outside of a circle with horses going in either direction.

### Effect of video angle on interobserver agreement

Fleiss’ kappa was used to examine interobserver agreement of subjective lameness between the 3 observers at each video angle (Table 1). When subjective lameness scores were considered as a score from 0 to 5 to account for degree of lameness, interobserver agreement for horses in the “lame” trials was moderate for video angle 2, fair for video angles 3, 4, 5, and 6, slight for video angles 1, 8, and 9, and poor for video angle 7 (Table 1a). When lameness was evaluated as a binary outcome, interobserver agreement was perfect for video angles 2 and 9, and poor to slight for all other angles (Table 2b). When subjective lameness scores were considered as a score from 0 to 5, interobserver agreement for horses in the “sound” trials was perfect for video angle 5, moderate for video angle 9, slight for video angles 1, 2, 3, and 4, and poor for video angles 6, 7 and 8. When subjective lameness scores were considered as a binary outcome, interobserver agreement for horses in the “sound” trials was perfect for video angle 5, moderate for video angles 2 and 9, slight for video angles 3 and 4, and poor for video angles 1, 6, 7 and 8. Overall, of the 9 video angles evaluated, video angle 2 in which horses were trotting directly towards the camera, had the best interobserver agreement for both categorizing a horse as lame or sound and for scoring the degree of lameness in horses with induced lameness. For “sound” trials, video angle 5 had the best interobserver agreement for both categorizing a horse as lame or sound and for scoring the degree of lameness in horses.

**Table 1:** A) Agreement between 3 expert observers using a 5-point lameness scale where 0/5 was considered to be sound and any grade from 1–5/5 was considered to be lame

Video Angle	Lame		Sound	
	Percent Agreement	Fleiss Kappa (*P* value)	Percent Agreement	Fleiss Kappa (*P* value)
1	0	0.009 (0.953)	50	0.2 (0.396)
2	50	0.413 (0.008)	83.3	0.182 (0.321)
3	33	0.283 (0.055)	50	0.169 (0.473)
4	16.7	0.204 (0.153)	66.7	0.2 (0.396)
5	16.7	0.204 (0.167)	100	1 (< 0.001)
6	33.3	0.224 (0.114)	50	-0.2 (0.396)
7	0	-0.0935 (0.554)	66.7	-0.125 (0.596)
8	16.7	0.159 (0.314)	66.7	-0.125 (0.596)
9	16.7	0.091 (0.604)	83.3	0.437 (0.063)

**Table 2:** B) Agreement between 3 expert observers with lame and sound considered as binary outcomes

Video Angle	Lame		Sound	
	Percent Agreement	Fleiss Kappa (*P* value)	Percent Agreement	Fleiss Kappa (*P* value)
1	83.3	-0.058 (0.803)	50	-0.2 (0.396)
2	100	1 (< 0.001)	83.3	0.437 (0.063)
3	50	-0.2 (0.396)	50	0.169 (0.473)
4	66.7	0.2 (0.396)	66.7	0.2 (0.396)
5	66.7	-0.125 (0.596)	100	1 (< 0.001)
6	50	-0.2 (0.396)	50	-0.2 (0.396)
7	66.7	-0.125 (0.596)	66.7	-0.125 (0.596)
8	66.7	0.2 (0.396)	66.7	-0.125 (0.596)
9	100	1 (< 0.001)	83.3	0.437 (0.063)

### Ease of grading

The median and interquartile range (IQR), describing how easy the 3 observers found it to score lameness for the different video angles, is shown in Table 3. There were no statistically significant differences in ease of grading score between the video angles. Interobserver agreement for ease of grading score was slight to poor for all video angles (Table 4).

**Table 3:** Median and interquartile range (IQR) of ease of grading by video angle for 3 observers. 1 = Very easy to grade; 5 = Very difficult to grade

Video Angle	Median	IQR
1	2	1, 3.25
2	2	1, 3
3	2	2, 3
4	2	1, 3
5	2	1.75, 3
6	2	2, 3
7	3	2, 3
8	2	1.75, 3
9	3	1.75, 3

**Table 4:** Interobserver agreement for ease of grading by video angle

Video Angle	Percent Agreement	Fleiss Kappa (*P* Value)
1	16.7	0.178 (0.061)
2	0	-0.082 (0.445)
3	8.33	-0.032 (0.768)
4	8.33	0.002 (0.984)
5	8.33	0.119 (0.232)
6	8.33	0.02 (0.849)
7	8.33	-0.056 (0.588)
8	0	-0.09 (0.359)
9	0	-0.079 (0.425)

## Discussion

There are many variables that affect the accuracy of subjective lameness assessments as they rely heavily on the visual evaluation skills of practitioners to detect asymmetry in movement [10]. Due to the increased prevalence of telemedicine in equine practice, we aimed to examine the effect of 9 different video angles on assessment of induced forelimb lameness. We included video recordings of horses trotting directly away and towards the video camera, horses trotting away and towards a video camera placed off to the left and right side, and horses trotting in a circle with the video camera placed on the inside and outside of a circle. These video angles were selected to mimic common video angles exchanged between horse owners, trainers, and equine veterinarians with the goal of determining the ideal video angle that should be used for collecting and assessing lameness information. We found that interobserver agreement for lameness degree in horses in the “lame” trials, i.e., with lameness induced, was moderate for angle 2 and poor to fair for all other angles. When lameness was considered as a binary outcome for the “lame” trials, interobserver agreement was perfect for angle 2 and 9 but remained poor to slight for all other angles. Interestingly, in the “sound” trials, the video angles with higher interobserver agreement were different than in the “lame” trials. Interobserver agreement was perfect for video angle 5 and moderate for video angle 9 when lameness degree was considered. When lameness was considered as a binary outcome, interobserver agreement was perfect for angle 5 and moderate for video angles 2 and 9. Overall, video angle 2 in which horses were trotting straight towards the video camera appeared to be the best video angle for assessing horses with forelimb lameness.

Subjective assessment of forelimb lameness is dependent on evaluating the symmetry of head and neck movement [1, 11]. Horses with unilateral forelimb lameness can be identified through asymmetric elevation of the head and neck as the lame limb hits the ground and initiates the stance phase. Considering that forelimb lameness is often associated with reliable asymmetry of head movement during trotting, it intuitively follows that videos of horses trotting directly toward the camera would be the best angle for gait assessment. We found that videos in which the horse only trotted directly towards the camera led to improved agreement among the 3 observers compared to videos in which the horse trotted both away and towards the camera. It is possible that this is because observers found assessment of head movement more challenging in horses trotting away from the camera which affected their overall assessment of the horse’s lameness. A previous study found that lameness evaluation of horses trotting in a circle is subject to widely variable visual approaches, instead of observers consistently evaluating head movement, which may affect lameness assessment between observers [11]. This could explain why the video angle with horses trotting directly towards the camera was better for lameness assessment than video angles in which horses were circling. That being said, subjective lameness scores were significantly different in lame horses versus sound horses for all video angles (Fig. 1). Interestingly, objective lameness data generated from horses trotting in a circle did have increased variability and the vector sum, HDmin, and HDmax were not significantly different between sound and lame horses in video angles 9, 9, and 8 and 9 respectively. The effect of circling on movement symmetry and objective lameness parameters have been demonstrated [12, 13]. Circling changes the gravitational and centripetal forces on the horse; therefore, the horse will lean into the center of the circle creating slightly asymmetric movement and alterations in data generated by inertial sensors [12, 13]. Finally, although limb did not affect lameness detection, this may have contributed to alterations in objective lameness parameters depending on whether the lame limb was on the inside or outside of the circle.

Previous studies have demonstrated that subjective assessment of lameness, especially in horses with mild lameness, is not reliable [14, 15]. This trend is amplified when comparing the reliability of forelimb and hindlimb lameness assessments, with the latter consistently identified as having low interobserver reliability [10, 14, 16, 17]. In this study, we found that interobserver agreement was affected by both video angle and whether the video represented a “sound” or “lame” trial. For horses with induced lameness, interobserver agreement was moderate (lameness scored 0–5) to excellent (binary outcome) for video angle 2 when horses were trotting directly towards the video camera. However, for the majority of other angles including observing horses trotting on a circle or observing horses from the side, interobserver agreement was low. This could suggest that these video angles make it more difficult for observers to score lameness. Interestingly, for horses in the “sound” trials, interobserver agreement was best for video angle 5 in which horses were trotting away and towards the video camera with the video camera placed 10 m to the right of the starting position. It is difficult to determine why interobserver agreement was different for horses in these trials, however, it is possible that despite being in the “sound” trial, these horses did have mild, subtle gait asymmetries that led to variability in subjective scoring among the observers. Additionally, with only 3 observers, slight changes in lameness scoring will greatly affect overall agreement. In general, interobserver agreement was higher when lameness was considered as a binary outcome and did not have to be more subtlety categorized by degree of lameness, regardless of whether horses were evaluated on a straight line or on a circle. Perhaps video recordings would be better suited for determining whether a horse is lame or sound, not determining the degree of lameness. Subtle changes in degree of lameness may be better assessed in person such that the veterinarian can observe the horse on several different surfaces, on straight lines and on circles, and can also use sound to assess gait.

The observers were also asked to subjectively score the ease of grading of each video angle for every trial. Although this was a subjective measurement and very open to reviewer interpretation, we aimed to include examination of how the different angles affected the ability of an individual reviewer to make an assessment. Although no significant differences were noted, angle 7 and angle 9 both had the highest (worst) median score [3] for ease of grading compared to all other angles that had a median score of 2. Both angles are obtained with horses trotting in a circle; therefore, it is possible that observers had more difficulty assessing the videos for lameness when horses were trotting in circles versus straight lines. Interobserver variability for ease of grading was poor across all video angles suggesting that there is a difference in how observers assess videos and formulate decisions about lameness.

Benefits of video recordings include documentation of clinical information, the ability to share lameness examinations amongst several people, and the ability to remotely assess horses when indicated, for example during preliminary pre-purchase examinations to determine if any obvious lameness is present that would preclude further examination of the horse. That being said, the authors strongly believe that live lameness examinations provide invaluable information that is more difficult to obtain from video recordings including the ability to evaluate the horse at variable speeds, incorporate sound, and perform musculoskeletal palpation, and should not be replaced by video recordings. Additionally, all videos in this study were viewed on a large computer monitor and if video recordings are to be evaluated, we would caution against viewing them on low-grade monitors or small screens, such as a phone, as this could decrease the reliability of the assessment.

Due to the questionable reliability of subjective lameness examinations, there have been efforts to produce more objective methods for lameness detection and quantification. Currently, the most commonly used system in clinical practice is a body-mounted inertial sensor system that detects asymmetries in head and pelvic movement [2, 18]. The present study utilized the Equinosis Q with Lameness Locator® software (Equinosis LLC, Columbia, MO) for objective lameness detection as described in previous studies [19]. While previous studies have reported moderate agreement between objective lameness parameters and subjective lameness scores [15, 19], we found excellent agreement between these parameters meaning that observers were consistently able to visually detect front limb gait asymmetry when it was present. This difference could be associated with the variable expertise of observers used in studies, differences in lameness scoring systems, or the inclusion of front limb versus hind lameness in the study.

Several limitations are present in this study including the use of horses with experimentally induced foot lameness. Although the use of solar pressure is a well-accepted model of experimental lameness, video recording of horses with naturally-occurring forelimb lameness may yield different results. Only mild-moderate forelimb lameness was induced in this study, therefore, assessment of horses with moderate or severe forelimb lameness may be different. Additionally, only forelimb lameness was assessed and the results cannot be extrapolated to hindlimb lameness. All sound and lame trials at the 9 different video angles were performed in the same order on each horse which could introduce some experimental bias if the horses’ lameness either improved or worsened with time. Additionally, an average of 22 strides were analyzed using the Equinosis Q with Lameness Locator® software due to the short lengths of each trial. The Equinosis Q with Lameness Locator® software recommends that a minimum of 25 strides in a straight line and 40 strides in a circle be analyzed, therefore, increased variability in the objective lameness data is likely. All trials were performed on hard, flat footing, therefore, cannot necessarily be extrapolated to other types of footing. No video recordings were obtained from the side, horses were trotted in circles in hand and not lunged, and standardization of speed was not possible in this field-based study, all of which can affect lameness and its assessment. No repeatability studies were performed to assess the consistency of gait before or after lameness induction and no intraobserver repeatability studies were performed. The sample size (*n* = 6) was small and yields lower statistical power. Finally, although Fleiss kappa is the statistical test of choice for evaluating interobserver agreement, given the small sample size, percent agreement may be more reflective of interobserver agreement and subjectively, two of the observers scored lameness more similarly than the third observer, which may have skewed the data to some degree.

## Conclusion

In conclusion, this study suggests that horses being remotely evaluated for forelimb lameness should have a video recording of the horse trotting directly towards the camera at a minimum as this is a reliable angle by which veterinarians can accurately assess lameness. Other angles can further contribute to assessment of the horse and can be provided in addition to the aforementioned video angle. This information can help standardize and improve remote assessment of horses as telemedicine is incorporated more frequently into the practice of forelimb lameness evaluation.

## Materials and methods

### Horses

Six University owned horses between the age of 3 and 13 years with a median age of 9.5 years were used in this study following approval by the Institutional Animal Care and Use Committee. There were 4 Thoroughbreds, 1 Quarter Horse and 1 Standardbred. Three horses were mares and 3 horses were geldings. All horses were maintained on pasture and were not in regular work at the time of the study. Prior to enrollment, horses were trotted in hand on hard ground in a straight line and in a circle in both directions. Horses were excluded if lameness was subjectively detected in any of the limbs as determined by an equine veterinarian board-certified in surgery and sports medicine and rehabilitation (KFO). Horses that paced instead of trotted were also excluded. Horses were maintained in a box stall when they were not being video recorded.

### Lameness induction

Lameness was induced with the use of a specialized horseshoe as previously described [19, 20]. Briefly, stainless steel shoes were nailed on to both front feet immediately prior to their trials (horses were normally maintained barefoot). The shoes had a 3/8” (9.53 mm) nut welded along the medial branch at the level of the apex of the frog which allowed insertion of a 3/8” (9.53 mm) cone screw that applied solar pressure to the medial bar. Screws were tightened to induce a mild-moderate but consistent, visually obvious unilateral forelimb lameness that was detected when trotting in a straight line. The limb for lameness induction was assigned using a random number generator (Excel®, Microsoft, Redmond, WA) with left front limb lameness induced in 3 horses and right front limb lameness induced in 3 horses. All horses were video recorded after application of both front shoes and prior to insertion of the cone screw to generate “sound” baseline trials. Video recording of horses for “lame” trials was performed immediately following insertion and tightening of the cone screw that was confirmed to generate mild-moderate but consistent, visually obvious unilateral forelimb lameness while trotting in a straight line.

### Video recordings

**Fig. 2:**
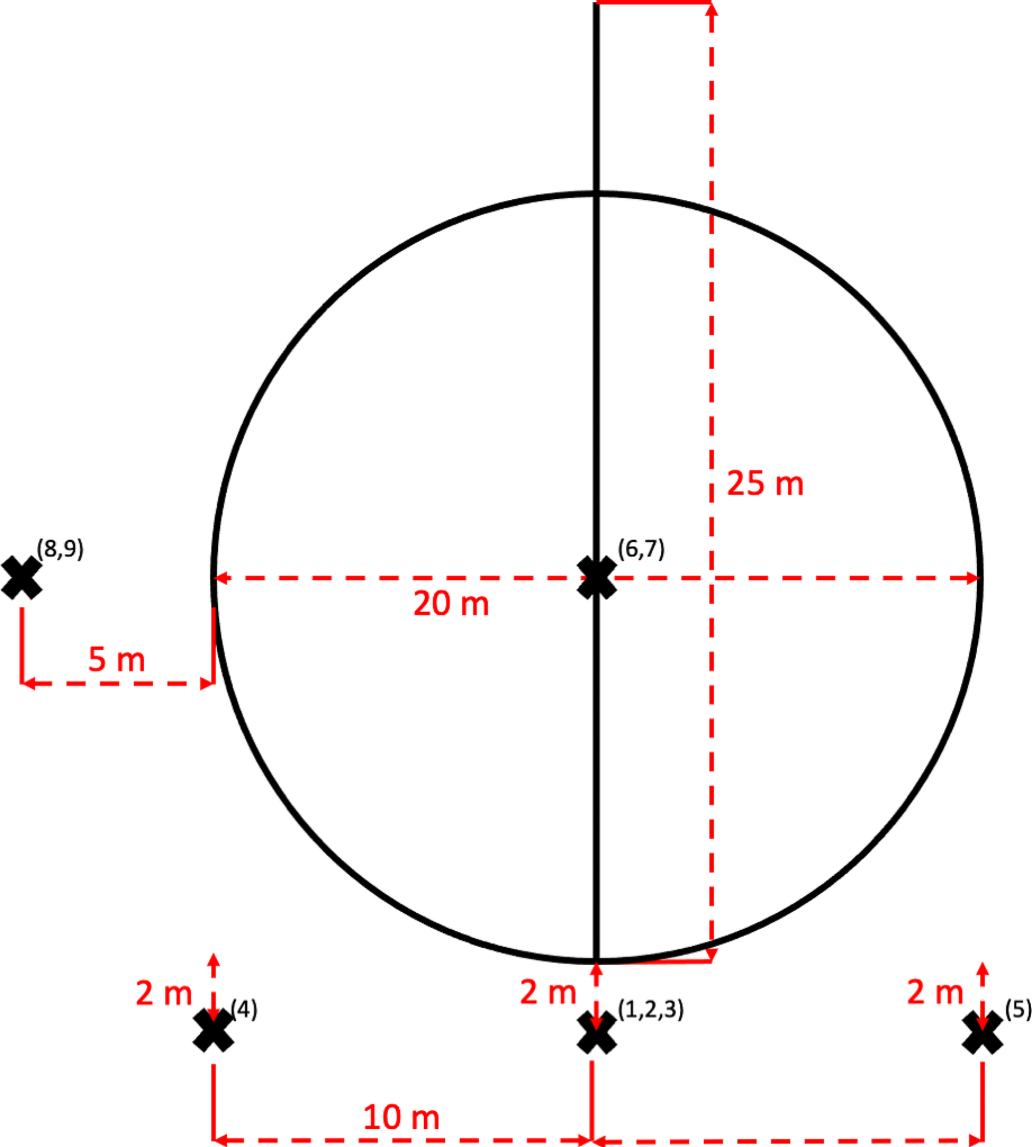
Schematic of study design and video angles including position of the camera (X), a 20 m circle and 25 m straight line. The video angles evaluated included: (1) horse trotting away 25 m from the video camera with the video camera directly behind the horse, (2) horse trotting 25 m towards the video camera with the video camera directly in front of the horse, (3) horse 25 m trotting away and towards the video camera with the video camera directly in front of the horse, (4) horse trotting away and towards the video camera for 25 m with the video camera placed 10 m to the left of the starting position, (5) horse trotting away and towards the video camera for 25 m with the video camera placed 10 m to the right of the starting position, (6) horse trotting in a 20 m circle to the left with the video camera placed in the center of the circle, (7) horse trotting in a 20 m circle to the right with the video camera placed in the center of the circle, (8) horse trotting in a 20 m circle to the left with the video camera placed 5 m to the outside of the circle, (9) horse trotting in a 20 m circle to the right with the video camera placed 5 m to the outside of the circle. For angles 1, 2, 3, 4 and 5, the video camera was placed 2 m in behind the starting position or 2 m in front of the ending position

All video recordings were obtained with an iPhone 12 camera (Apple, Cupertino, CA) mounted on a 62” (157.5 cm) tripod stand that could be rotated 360 degrees (Aureday, Shenzhen, China). All videos were captured in landscape mode and without any zoom. All trials were conducted on the same, flat asphalt surface over 2 days with the same environmental conditions on both days. The same handler was used for all trials on a single horse. Horses were trotted at a moderate speed in hand with the same handler on the left side of the horse for all trials. Two cones were placed 25 m apart to ensure all horses would trot in a straight line for the same distance that would be filmed from the same angle. Similarly, a 20 m diameter circle was traced using chalk to ensure all horses were trotted in the same circular path. All video camera locations were also marked by chalk to ensure consistency. For trials performed on a circle, the video camera was centered on the horse and followed the horse as it trotted. A single, complete circle was captured. There were 9 video angles obtained in the study including: (1) horse trotting away from the video camera with the video camera directly behind the horse (straight away), (2) horse trotting towards the video camera with the video camera directly in front of the horse (straight towards), (3) horse trotting away and towards the video camera with the video camera directly in front of the horse (straight away and towards), (4) horse trotting away and towards the video camera with the video camera placed 10 m to the left of the starting position (left away and towards), (5) horse trotting away and towards the video camera with the video camera placed 10 m to the right of the starting position (right away and towards), (6) horse trotting in a circle to the left with the video camera placed in the center of the circle (inside left circle), (7) horse trotting in a circle to the right with the video camera placed in the center of the circle (inside right circle), (8) horse trotting in a circle to the left with the video camera placed 5 m to the outside of the circle (outside left circle), (9) horse trotting in a circle to the right with the video camera placed 5 m to the outside of the circle (outside right circle) (Fig. 2). For video angles 1–3, the video camera was 2 m behind the starting position. For video angles 4 and 5, the horse’s entire line of trot was captured from where the video camera was placed. All horses completed all 9 video angles in the order listed above for the “sound” trial and then immediately completed all 9 video angles in the order listed above for the “lame” trial.

A total of 108 videos were obtained and saved as MOV files. Video recordings of each trial were randomized using a random list generator (Excel®, Microsoft, Redmond, WA). Audio was removed from the videos as clips contained audio indicating the horse’s name and soundness status. Once randomized, the video recordings designated with a number were compiled into a single file using a video editing software (iMovie, Apple, Cupertino, CA). Randomization and editing were performed by an author (APV) that was not involved in lameness assessment.

### Subjective lameness assessment

The video recordings were reviewed by three blinded equine veterinarians board-certified in equine sports medicine and rehabilitation and experienced in equine lameness assessment (KFO, KAB, EJD). All videos were viewed on a computer screen (32 inches (81 cm) x 14 inches (35 cm)). Reviewers were allowed to speed up or slow down videos as needed and were allowed to review the video as many times as they felt was necessary to subjectively grade lameness. Each individual trial was given a score from 0 to 5 by each reviewer using the subjective lameness scale previously described by Ross with slight modifications (Table 5) [1]. Individual lameness scores for each reviewer are provided in Supplementary Table 1. A median lameness score was calculated using the reviewers scores for each individual trial and then the median lameness score for each video angle was calculated for the 6 horses. Reviewers were also asked to score each video for subjective ease of grading where 1 = very easy to grade lameness, 2 = easy to grade lameness, 3 = neither easy or difficult to grade lameness, 4 = difficult to grade lameness, and 5 = extremely difficult to grade lameness.

**Table 5:** Subjective lameness scoring system for front limb lameness in horses trotting on a hard surface as by Ross in Diagnosis and Management of Lameness in the Horse, 2nd Edition [1]

Lameness score	Observations
0	Sound
1	Mild lameness observed while the horse is trotted in a straight line. When the lame forelimb strikes, a subtle head nod is observed. The head nod may be inconsistent at times.
2	Obvious lameness is observed. The head nod is seen consistently and excursion is several centimeters.
3	Pronounced head nod of approximately 50% or more centimeters than observed in horses with lameness score of 2.
4	Severe lameness with extreme head nod is present. The horse can still be trotted, however.
5	The horse does not bear weight on the limb. If trotted, the horse carries the limb.

### Objective lameness assessment

Horses were fitted with body-mounted inertial sensors (Equinosis Q with Lameness Locator® software, Equinosis LLC, Columbia, MO) for the duration of the video recording trials. Sensors were placed according to the manufacturer’s directions including a poll sensor secured to a head piece attached to the halter, a right front limb sensor attached to the dorsal surface of the pastern, and a pelvic sensor secured to an adhesive patch applied on the dorsal midline over both tuber sacrale. Data was obtained wirelessly using the Equinosis Q with Lameness Locator® software package (Equinosis LLC, Columbia, MO) with default settings. The difference in minimum head position (HD_min_), difference in maximum head position (HD_max_), and a summary measure of head movement asymmetry (vector sum, VS) were recorded for every individual trial. Positive HD_min_ and HD_max_ values are associated with right forelimb lameness, while negative HD_min_ and HD_max_ values are associated with left forelimb lameness. Therefore, values obtained from horse with a left forelimb lameness were multiplied by -1 for further analysis. Median and interquartile range were calculated for each “sound” and “lame” video angle.

### Statistical analysis

Analyses were conducted with Stata 14.1MP (StataCorp, College Station TX) and R software (version 4.2.2) in RStudio (version 2023.06.0 + 421), with two-sided tests of hypotheses and a p-value < 0.05 as the criterion for statistical significance. All data was evaluated for normality when appropriate using the Shapiro-Wilk test. Multivariable mixed effects logistic regression was used to examine the success of lameness induction with all “sound” and “lame” trials at all video angles considered together. *Post-hoc* estimation, the area under the curve (AUC) of the receiver operating characteristics (ROC) was calculated. The OR were reported with their respective 95% confidence intervals (95% CI). Descriptive statistics (median and interquartile range (IQR) for ease of grading by video angle were calculated, while Fleiss kappa was to evaluate interobserver agreement between the 3 observers using the irr package. For Fleiss’s kappa statistic, values < 0.00 were considered to be poor agreement, values 0.00–0.20 indicated slight agreement, values 0.21–0.40 indicated fair agreement, values 0.41– 0.60 indicated moderate agreement, values 0.61–0.80 indicated substantial agreement, and values 0.81–1.00 indicated almost perfect agreement [21].

### Electronic supplementary material

Below is the link to the electronic supplementary material.

Supplementary Material 1

Supplementary Material 2

## Data Availability

The datasets used and/or analysed during the current study are available from the corresponding author on reasonable request.

## Abbreviations

AUC: Area under the curve
CI: Confidence interval
HD_max_: Difference in head maximum heights
HD_min_: Difference in head minimum heights
κ: Kappa
IQR: Interquartile range
OR: Odds ratio
PPE: Prepurchase examinations
ROC: Receiver operating characteristics
VS: Vector sum
