# Supplementary material for: Effect of video angle on detection of induced front limb lameness in horses
Source: BMC Vet Res. 2024 May 3;20:172. doi: 10.1186/s12917-024-04032-9 (PMC11067204; doi:10.1186/s12917-024-04032-9)
Supplement: Supplementary file 1 — Supplementary Material 1 [file 12917_2024_4032_MOESM1_ESM.docx]

**Supplemental Table 1.** Subjective lameness scores of three reviewers scoring videos obtained at 9 different video angles in horses with lameness induced (lame) or no lameness induced (sound).

| **Horse** | **Video Angle** | **Lameness Induced** | **Reviewer #1 Lameness Grade** | **Reviewer #2 Lameness Grade** | **Reviewer #3 Lameness Grade** |
| --- | --- | --- | --- | --- | --- |
| 1 | 1 | Lame | 3 | 2 | 2 |
| 2 | 1 | Lame | 3 | 2 | 2 |
| 3 | 1 | Lame | 2 | 2 | 1 |
| 4 | 1 | Lame | 3 | 3 | 2 |
| 5 | 1 | Lame | 1 | 1 | 0 |
| 6 | 1 | Lame | 2 | 1 | 1 |
| 1 | 1 | Sound | 1 | 0 | 0 |
| 2 | 1 | Sound | 1 | 0 | 0 |
| 3 | 1 | Sound | 0 | 0 | 0 |
| 4 | 1 | Sound | 0 | 0 | 0 |
| 5 | 1 | Sound | 1 | 0 | 0 |
| 6 | 1 | Sound | 0 | 0 | 0 |
| 1 | 2 | Lame | 3 | 3 | 2 |
| 2 | 2 | Lame | 3 | 2 | 2 |
| 3 | 2 | Lame | 2 | 2 | 2 |
| 4 | 2 | Lame | 2 | 2 | 2 |
| 5 | 2 | Lame | 0 | 0 | 0 |
| 6 | 2 | Lame | 2 | 1 | 2 |
| 1 | 2 | Sound | 0 | 0 | 0 |
| 2 | 2 | Sound | 0 | 0 | 0 |
| 3 | 2 | Sound | 0 | 0 | 0 |
| 4 | 2 | Sound | 0 | 0 | 0 |
| 5 | 2 | Sound | 2 | 1 | 0 |
| 6 | 2 | Sound | 0 | 0 | 0 |
| 1 | 3 | Lame | 2 | 2 | 0 |
| 2 | 3 | Lame | 2 | 2 | 2 |
| 3 | 3 | Lame | 1 | 1 | 0 |
| 4 | 3 | Lame | 3 | 3 | 2 |
| 5 | 3 | Lame | 2 | 1 | 0 |
| 6 | 3 | Lame | 1 | 1 | 1 |
| 1 | 3 | Sound | 1 | 1 | 0 |
| 2 | 3 | Sound | 1 | 1 | 0 |
| 3 | 3 | Sound | 0 | 0 | 0 |
| 4 | 3 | Sound | 0 | 0 | 0 |
| 5 | 3 | Sound | 1 | 0 | 0 |
| 6 | 3 | Sound | 0 | 0 | 0 |
| 1 | 4 | Lame | 3 | 2 | 2 |
| 2 | 4 | Lame | 3 | 2 | 2 |
| 3 | 4 | Lame | 2 | 2 | 0 |
| 4 | 4 | Lame | 3 | 2 | 2 |
| 5 | 4 | Lame | 1 | 1 | 1 |
| 6 | 4 | Lame | 1 | 0 | 0 |
| 1 | 4 | Sound | 1 | 1 | 0 |
| 2 | 4 | Sound | 0 | 0 | 0 |
| 3 | 4 | Sound | 0 | 1 | 0 |
| 4 | 4 | Sound | 0 | 0 | 0 |
| 5 | 4 | Sound | 0 | 0 | 0 |
| 6 | 4 | Sound | 0 | 0 | 0 |
| 1 | 5 | Lame | 3 | 3 | 2 |
| 2 | 5 | Lame | 3 | 2 | 2 |
| 3 | 5 | Lame | 2 | 1 | 1 |
| 4 | 5 | Lame | 2 | 2 | 2 |
| 5 | 5 | Lame | 1 | 1 | 0 |
| 6 | 5 | Lame | 1 | 1 | 0 |
| 1 | 5 | Sound | 0 | 0 | 0 |
| 2 | 5 | Sound | 0 | 0 | 0 |
| 3 | 5 | Sound | 0 | 0 | 0 |
| 4 | 5 | Sound | 0 | 0 | 0 |
| 5 | 5 | Sound | 0 | 0 | 0 |
| 6 | 5 | Sound | 0 | 0 | 0 |
| 1 | 6 | Lame | 3 | 2 | 1 |
| 2 | 6 | Lame | 2 | 2 | 2 |
| 3 | 6 | Lame | 1 | 1 | 1 |
| 4 | 6 | Lame | 3 | 3 | 0 |
| 5 | 6 | Lame | 2 | 1 | 0 |
| 6 | 6 | Lame | 1 | 1 | 0 |
| 1 | 6 | Sound | 1 | 0 | 0 |
| 2 | 6 | Sound | 0 | 0 | 0 |
| 3 | 6 | Sound | 1 | 0 | 0 |
| 4 | 6 | Sound | 1 | 0 | 0 |
| 5 | 6 | Sound | 0 | 0 | 0 |
| 6 | 6 | Sound | 0 | 0 | 0 |
| 1 | 7 | Lame | 3 | 3 | 2 |
| 2 | 7 | Lame | 3 | 2 | 2 |
| 3 | 7 | Lame | 2 | 1 | 1 |
| 4 | 7 | Lame | 2 | 1 | 1 |
| 5 | 7 | Lame | 2 | 1 | 0 |
| 6 | 7 | Lame | 2 | 2 | 1 |
| 1 | 7 | Sound | 0 | 0 | 0 |
| 2 | 7 | Sound | 0 | 0 | 0 |
| 3 | 7 | Sound | 0 | 0 | 0 |
| 4 | 7 | Sound | 0 | 0 | 0 |
| 5 | 7 | Sound | 1 | 0 | 0 |
| 6 | 7 | Sound | 0 | 1 | 0 |
| 1 | 8 | Lame | 2 | 1 | 1 |
| 2 | 8 | Lame | 2 | 2 | 2 |
| 3 | 8 | Lame | 1 | 1 | 0 |
| 4 | 8 | Lame | 3 | 2 | 2 |
| 5 | 8 | Lame | 2 | 2 | 1 |
| 6 | 8 | Lame | 0 | 1 | 0 |
| 1 | 8 | Sound | 1 | 0 | 0 |
| 2 | 8 | Sound | 0 | 0 | 0 |
| 3 | 8 | Sound | 1 | 0 | 0 |
| 4 | 8 | Sound | 0 | 0 | 0 |
| 5 | 8 | Sound | 0 | 0 | 0 |
| 6 | 8 | Sound | 0 | 0 | 0 |
| 1 | 9 | Lame | 3 | 3 | 2 |
| 2 | 9 | Lame | 3 | 2 | 2 |
| 3 | 9 | Lame | 1 | 1 | 1 |
| 4 | 9 | Lame | 2 | 1 | 1 |
| 5 | 9 | Lame | 2 | 1 | 1 |
| 6 | 9 | Lame | 2 | 1 | 1 |
| 1 | 9 | Sound | 0 | 0 | 0 |
| 2 | 9 | Sound | 0 | 0 | 0 |
| 3 | 9 | Sound | 0 | 0 | 0 |
| 4 | 9 | Sound | 0 | 0 | 0 |
| 5 | 9 | Sound | 0 | 0 | 0 |
| 6 | 9 | Sound | 1 | 1 | 0 |
